# Cerium promoted V-g-C_3_N_4_ as highly efficient heterogeneous catalysts for the direct benzene hydroxylation

**DOI:** 10.1098/rsos.180371

**Published:** 2018-06-27

**Authors:** Cheng Wang, Liya Hu, Meiyin Wang, Bin Yue, Heyong He

**Affiliations:** Department of Chemistry and Shanghai Key Laboratory of Molecular Catalysis and Innovative Materials, Collaborative Innovation Center of Chemistry for Energy Materials, Fudan University, Shanghai 200433, China

**Keywords:** carbon nitride, cerium, vanadium, benzene hydroxylation

## Abstract

A series of Ce_*x*_-V-g-C_3_N_4_ catalysts with different cerium content were synthesized by a facile co-assembly method. Compared with pure V-g-C_3_N_4_ catalyst, the addition of cerium facilitated the high dispersion of vanadium species as well as the benzene adsorption ability of the corresponding catalysts. Also, the existence of cerium promoted the partial reduction of vanadium species, which improved the redox property of vanadium species as the active centres. The Ce_*x*_-V-g-C_3_N_4_ catalysts showed considerably improved activity in the benzene hydroxylation reaction compared with V-g-C_3_N_4_ catalyst. Among the catalysts studied, Ce_0.07_-0.07 V-g-C_3_N_4_ exhibited the best catalytic activity with a benzene conversion of 33.7% and a phenol yield of 32.3% with good structural and catalytic stability, while only 24.7% of benzene conversion and phenol yield of 24.2% were obtained over 0.07 V-g-C_3_N_4_.

## 1. Introduction

Phenol, as an important chemical intermediate in industry, is widely employed in the synthesis of aniline, resins, plastics, bactericides and agrochemicals [1]. However, the current phenol production is based upon the three-step cumene process, which has some inevitable disadvantages, e.g. the by-product acetone with low market demand, the complex synthesis steps and the high energy consumption [2]. From the view of green chemistry, the direct hydroxylation of benzene to phenol has attracted great interest in the past few decades. Since it is difficult to insert an oxygen atom into the stable C─H bond of benzene, many efforts have been devoted to searching for appropriate oxidants [3]. Among them, molecular oxygen, nitrous oxide and hydrogen peroxide are three main kinds of oxidants used in the benzene hydroxylation reaction [4–7]. However, molecular oxygen is too stable to be activated mildly, while nitrous oxide is not easily available in industry. In contrast, hydrogen peroxide shows superior properties with the mild reaction condition of benzene oxidation and water as the green by-product.

Up to now, catalysts with various metal species, such as V, Fe, Cu, Co and Ti [8–12], have been employed for this titled reaction. Among them, vanadium species exhibit excellent catalytic activity. As for the catalyst support, many metal oxides such as Al_2_O_3_, SiO_2_ and TiO_2_ were widely used [13–15]. However, the weak interaction between V species and the metal oxide support leads to undesirable loss of V active spices [16]. Besides, some metal oxide supports, such as Al_2_O_3_ and ZrO_2_, disfavour the redox cycle between V^5+^ and V^4+^ [17,18], leading to the low activity in the direct oxidation of benzene to phenol.

Nowadays, graphitic carbon nitride (g-C_3_N_4_), as an analogue of graphene, has been widely used as a catalyst support, due to its unique physico-chemical property and easily modified feature. Compared with pure graphene, the N-containing groups with strong coordination ability may facilitate the dispersion and stability of metal species. Moreover, carbon support will show certain reduction ability at high temperature, affecting the valence state of loaded metal species [19–21]. In previous work [22], we found that V^5+^ species were partially reduced to V^4+^ ones during the calcination process over g-C_3_N_4_ support. And the resulting V-g-C_3_N_4_ catalyst showed excellent catalytic performance and stability in the direct benzene hydroxylation. As is known, the transformation between V^5+^ and V^4+^ species plays an important role in the benzene conversion. Therefore, it is worth exploring the proper molar ratio of V^4+^/V^5+^ to obtain the most effective catalyst for the titled reaction.

Recently, bi-metal or multi-metal catalysts have shown competitive advantages in redox reactions. As the most abundant rare earth element, cerium is widely used in catalytic oxidation due to its unique electronic structures. The different electronic configurations between Ce^3+^ with 4f^1^5d^0^ and Ce^4+^ with 4f^0^5d^0^ lead to the formation of good redox couple of Ce^3+^/Ce^4+^ [23]. Paz *et al.* found that the addition of second metal cerium enhanced the interactions between Pt^0^ and oxygen atoms. As a result, the bi-metal catalyst showed higher activity in the CO oxidation reaction even at lower reaction temperature compared with the non-promoted Pt catalysts [24]. Lu *et al.* prepared a series of CeO_2_–Co_3_O_4_ catalysts for the catalytic oxidation of formaldehyde (HCHO) [25]. The unique redox property of Ce played a crucial role in the excellent performance in HCHO oxidation.

In this study, we added cerium as the second metal into V supported g-C_3_N_4_ in order to facilitate the redox property of V species. A series of cerium-doped V-g-C_3_N_4_ catalysts were prepared by a facile co-assembly method using vanadylacetylacetonate, cerium nitrate and melamine as precursors. All the Ce doped V-g-C_3_N_4_ catalysts showed higher activity than the mono-metal ones. The strong synergistic effects between Ce and V result in the excellent redox property of V species, which improves the catalytic performance of corresponding catalysts in benzene hydroxylation.

## 2. Material and methods

### 2.1. Synthesis

Melamine, vanadylacetylacetonate (C_10_H_14_O_5_V) and Ce(NO_3_)_3_·6H_2_O were all purchased from Aladdin Industrial Corporation and used without further purification. g-C_3_N_4_ was prepared by the direct calcination of melamine at 550°C for 2 h under nitrogen atmosphere [26]. 0.07 V-g-C_3_N_4_ sample was obtained as follows: 0.42 g of C_10_H_14_O_5_V and 2.50 g of melamine were mixed with 50 ml of ethanol. After stirring vigorously at 50°C for 1 h, the solution was dried at 60°C overnight. The resulting solid sample was calcined in N_2_ from room temperature to 550°C with a heating rate of 2°C min^−1^ and kept at 550°C for another 2 h. After cooling to room temperature, the product was collected and denoted as 0.07 V-g-C_3_N_4_, where 0.07 represented the theoretical weight content of vanadium. 0.05 V-g-C_3_N_4_ and 0.10 V-g-C_3_N_4_ catalysts were prepared according to the same synthesis procedure as for 0.07 V-g-C_3_N_4_ by adding desired amount of C_10_H_14_O_5_V.

As for 0.07Ce-g-C_3_N_4_, the synthesis method was also similar to that of 0.07 V-g-C_3_N_4_, except for adding 0.23 g of Ce(NO_3_)_3_·6H_2_O rather than 0.42 g of C_10_H_14_O_5_V. Furthermore, by adding both 0.42 g of C_10_H_14_O_5_V and different amounts of Ce(NO_3_)_3_·6H_2_O, Ce_*x*_-0.07 V-g-C_3_N_4_ catalysts were obtained, where *x* represents the molar ratio of Ce/V.

### 2.2. Benzene hydroxylation reaction

The direct benzene hydroxylation reaction was conducted as follows. Typically, 1 ml of benzene, 10 ml of 80 wt% acetic acid and 40 mg of catalyst were added into a 25 ml three-necked flask connected with a reflux condenser. After heating to 70°C, 3.5 ml of 30 wt% H_2_O_2_ was added dropwise in 30 min with vigorously stirring. The reaction mixture was stirred for another 4 h. After reaction, the catalyst was separated by centrifugation and the content of liquid products was analysed immediately by gas chromatography using toluene as the internal standard.

### 2.3. Material characterization

Elemental analysis was performed with a Thermo Elemental IRIS Intrepid inductively coupled plasma-atomic emission spectrometer (ICP-AES). Transmission electron microscopic (TEM) images were acquired with a FEI Tecnai G^2^ F20 S-Twin field-emission transmission electron microscope operated at 200 kV. Elemental mapping was conducted using a Philips XL 30 microscope with energy dispersive X-ray spectrometer operated at 30 kV. Fourier transform infrared (FT-IR) spectra were recorded with a Nicolet iS10 infrared instrument using KBr discs. X-ray diffraction (XRD) patterns were recorded on a Bruker D8 Advances X-ray diffractometer using Cu-Kα radiation with a voltage of 40 kV and a current of 40 mA. X-ray photoelectron spectra (XPS) were recorded with a Perkin-Elmer PHI 5000C ESCA system equipped with a dual X-ray source by using Mg Kα (1253.6 eV) anode and a hemispherical energy analyser. Specific surface area results were obtained at 77 K using a Micromeritics Tristar 3000 apparatus. The benzene adsorption was measured using a Hiden intelligent gravimetric analyser. All samples were degassed under a vacuum of less than 10^−3^ Pa at 300°C for 6 h prior to the adsorption measurement.

## 3. Results and discussion

The results of benzene hydroxylation reaction over all catalysts studied are shown in table 1 and electronic supplementary material, table S1. By optimizing the vanadium content in the catalysts, the best catalytic activity in the benzene hydroxylation reaction was obtained over 0.07 V-g-C_3_N_4_ catalyst with 7 wt% of vanadium content. With the introduction of cerium into the 0.07 V-g-C_3_N_4_ catalyst (Ce/V = 0.05), the benzene conversion is improved slightly from 24.7% to 25.3% (entry 2, table 1). With the further rise of cerium content, the benzene conversion increases remarkably to the highest 33.7% over Ce_0.07_-0.07 V-g-C_3_N_4_ (entry 3, table 1) and then decreases to 29.4% over Ce_0.10_-0.07 V-g-C_3_N_4_ (entry 4, table 1), while the phenol selectivity remains almost constant with the variation of cerium content. Moreover, the carbon balance value shown in table 1 are greater than 99% over the three Ce-containing catalysts, indicating a high carbon yield with negligible side reactions. The TOF value of 17.1 h^−1^ based on the vanadium content also indicates that Ce_0.07_-0.07 V-g-C_3_N_4_ is the most active catalyst. Thus, we deduce that a proper molar ratio of Ce/V possibly facilitates both the redox properties of V species and the efficient decomposition of H_2_O_2_, leading to the remarkable catalytic activity in the benzene conversion reaction [27].

**Table 1.** Catalytic activity of various catalysts for benzene hydroxylation reaction. Reaction conditions: 1 ml of benzene, 10 ml of 80 wt% acetic acid, 40 mg of catalyst, 3.5 ml of 30 wt% H_2_O_2_, 70°C for 4 h.

							carbon out (mmol)			
entry	catalyst	benzene conv. (%)	phenol select. (%)	phenol yield (%)	TOF^a^ value (h^−1^)	carbon in (mmol)	unreacted benzene	phenol	by-products^b^	carbon balance closure^c^ (%)
1	0.07 V-g-C_3_N_4_	24.7	98.1	24.2	12.4	67.6	50.9	16.4	0.2	>99
2	Ce_0.05_-0.07 V-g-C_3_N_4_	25.3	96.3	24.4	12.7	67.6	50.5	16.5	0.5	>99
3	Ce_0.07_-0.07 V-g-C_3_N_4_	33.7	95.9	32.3	17.1	67.6	44.8	21.8	0.9	>99
4	Ce_0.10_-0.07 V-g-C_3_N_4_	29.4	97.6	28.7	15.1	67.6	47.7	19.4	0.5	>99

^a^Turnover frequency (TOF) was calculated as the molecules of generated phenol per metal atom per hour.

^b^The total amount of hydroquinone and catechol.

^c^The carbon balance closure was calculated as the molar ratio of carbon out to carbon in.

In order to elucidate the synergistic effects between V and Ce species, we selected g-C_3_N_4_, 0.07 V-g-C_3_N_4_ and Ce_0.07_-0.07 V-g-C_3_N_4_ as the representative catalysts to carry out detailed characterizations. Generally, the specific surface area of a catalyst has a certain influence on the catalytic activity. As shown in table 2, the specific surface area increases slightly after the introduction of metal species compared with that of the non-modified g-C_3_N_4_ catalyst (entries 1–3) and Ce_0.07_-0.07 V-g-C_3_N_4_ has the highest specific surface area. However, the quite different catalytic performances between 0.07 V-g-C_3_N_4_ and Ce_0.07_-0.07 V-g-C_3_N_4_ demonstrate that the specific surface area is not the main reason for the different catalytic activity.

**Table 2:** Specific surface area and metal contents of various catalysts.

entry	catalyst	*S*_BET_ (m^2^ g^−1^)	vanadium content (wt.%)^a^	cerium content (wt.%)^a^
1	g-C_3_N_4_	32	—	—
2	0.07 V-g-C_3_N_4_	43	7.0	—
3	Ce_0.07_-0.07 V-g-C_3_N_4_	47	6.8	0.5

^a^Analysed by ICP-AES.

**Figure 1:**
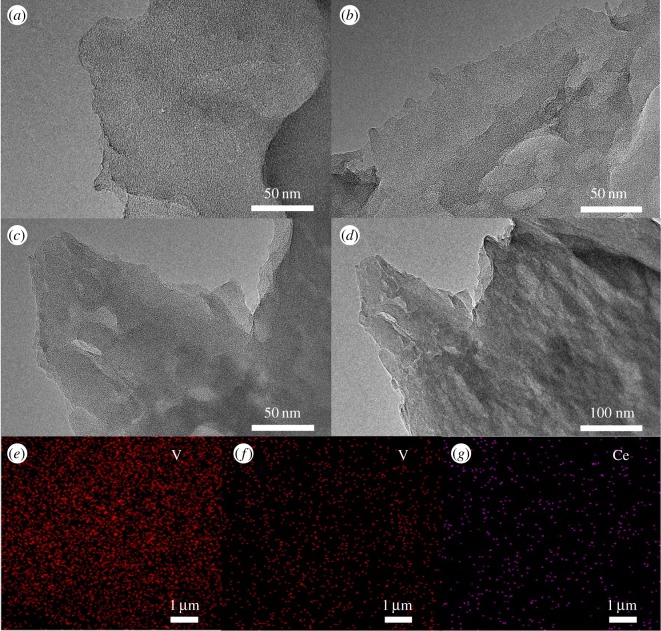
TEM images of (*a*) g-C_3_N_4_. (*b*) 0.07 V-g-C_3_N_4_ and (*c*,*d*) Ce_0.07_-0.07 V-g-C_3_N_4_. V mapping of (*e*) 0.07 V-g-C_3_N_4_ and (*f*) Ce_0.07_-0.07 V-g-C_3_N_4_ and Ce mapping of (*g*) Ce_0.07_-0.07 V-g-C_3_N_4_.

The morphologies of the catalysts were studied by TEM. As shown in figure 1*a*–*d*, all the samples exhibit the typical large lamellar structure, indicating that the structure of g-C_3_N_4_ support remains after the incorporation of metal species. As for 0.07 V-g-C_3_N_4_ and Ce_0.07_-0.07 V-g-C_3_N_4_ catalysts (figure 1*b*–*d*), no obvious metal particles are observed over both catalysts, implying the high dispersion of metal species. Also, the element-mapping images of 0.07 V-g-C_3_N_4_ and Ce_0.07_-0.07 V-g-C_3_N_4_ catalysts (figure 1*e*–*g*) further demonstrate that both V and Ce species are highly dispersed over the g-C_3_N_4_ support.

However, it is worth noting that the vanadium dispersion of 0.07 V-g-C_3_N_4_ is slightly poorer than that of Ce_0.07_-0.07 V-g-C_3_N_4_. This implies that the existence of cerium may facilitate the high dispersion of vanadium, leading to the high catalytic activity [28].

The graphitic stacking structures of the catalysts were also confirmed by XRD patterns. For pure g-C_3_N_4_ (electronic supplementary material, figure S1*a*), two distinct diffractions are observed at *ca* 13.2° and 27.5°, corresponding to (100) diffraction of in-planar repeating motifs of tris-*s*-triazine units and (002) diffraction of interlayer stacking aromatic systems, respectively [29]. After the addition of metal species, both corresponding (100) and (002) diffraction peaks become broader, suggesting that the ordered structure of g-C_3_N_4_ support decreases slightly. In addition, the (002) peaks of 0.07 V-g-C_3_N_4_ and Ce_0.07_-0.07 V-g-C_3_N_4_ catalysts both shift to low angle slightly compared with that of g-C_3_N_4_, indicating that the metal oxides were incorporated into the g-C_3_N_4_ sheets successfully [30]. Furthermore, no distinct peak originating from either vanadium or cerium species can be observed, which implies that the metal species are in non-crystallized state or dispersed well on the g-C_3_N_4_ layers. These results are consistent with the results of TEM.

**Figure 2.**
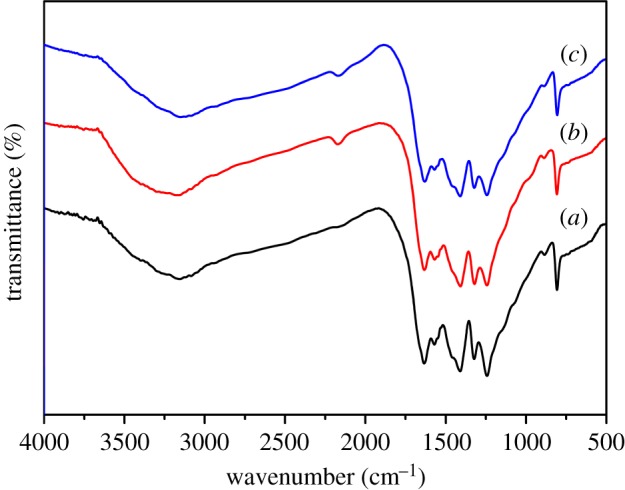
FT-IR spectra of (*a*) g-C_3_N_4_, (*b*) 0.07 V-g-C_3_N_4_ and (*c*) Ce_0.07_-0.07 V-g-C_3_N_4_.

The FT-IR spectra of g-C_3_N_4_, 0.07 V-g-C_3_N_4_ and Ce_0.07_-0.07 V-g-C_3_N_4_ catalysts are shown in figure 2. As for g-C_3_N_4_ (figure 2*a*), the major bands between 1200 and 1650 cm^−1^ are attributed to the stretching modes of CN heterocycles, while the band at 804 cm^−1^ corresponds to the stretching mode of triazine units (C_6_N_7_). The broad bands in the range of 3000–3400 cm^−1^ can be ascribed to the stretching of N─H bonds in both uncondensed amino groups and adsorbed water molecules [31]. For 0.07 V-g-C_3_N_4_ and Ce_0.07_-0.07 V-g-C_3_N_4_ (figure 2*b*,*c*), their spectra are similar to that of g-C_3_N_4_ except for a small additional band at 2157 cm^−1^ corresponding to the disturbance of conjugated N═C─N units after metal doping [32,33].

**Figure 3.**
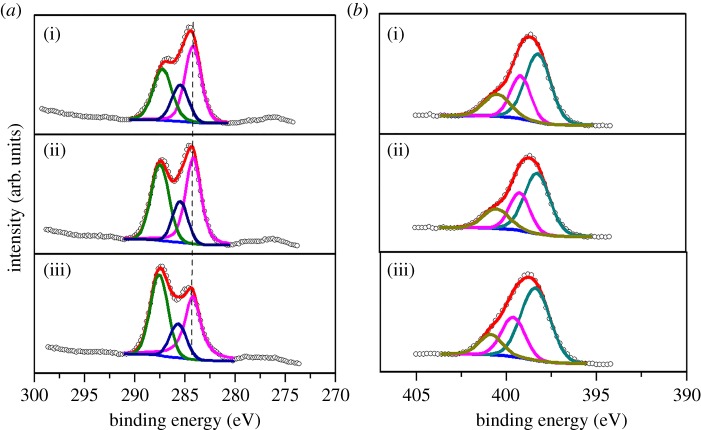
High resolution XPS spectra of C 1s (*a*) and N 1s (*b*) of (i) g-C_3_N_4_, (ii) 0.07 V-g-C_3_N_4_ and (iii) Ce_0.07_-0.07 V-g-C_3_N_4_.

**Figure 4.**
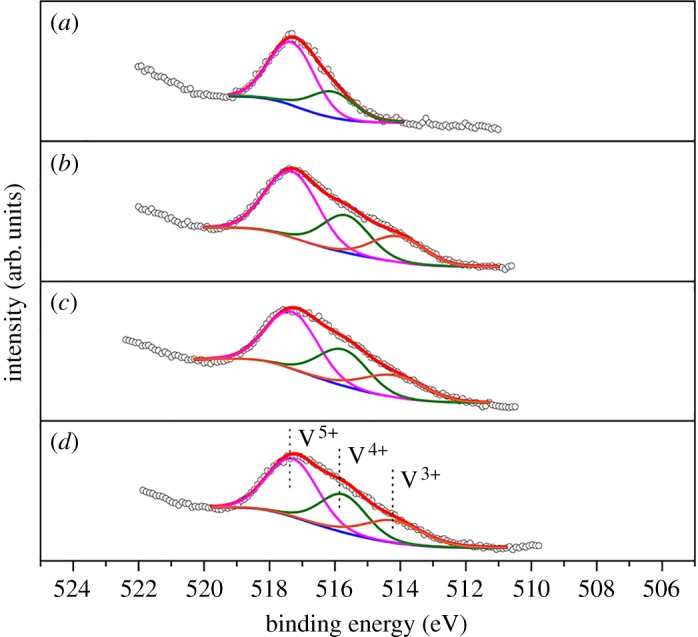
V 2p_3/2_ XPS spectra of (*a*) 0.07 V-g-C_3_N_4_, (*b*) Ce_0.05_-0.07 V-g-C_3_N_4_, (*c*) Ce_0.07_-0.07 V-g-C_3_N_4_ and (*d*) Ce_0.10_-0.07 V-g-C_3_N_4_.

In order to investigate the surface chemical composition of the catalysts, XPS measurement was carried out. As shown in electronic supplementary material, figure S2, C and N species, which refer to the peaks at binding energies of 288.0 (C 1s) and 400.0 (N 1s), are the main elements in all catalysts. By subtracting the peak area of the contaminant carbon C 1s at 284.6 eV, the peak area ratios of C 1s/N 1s of g-C_3_N_4_, 0.07 V-g-C_3_N_4_ and Ce_0.07_-0.07 V-g-C_3_N_4_ catalysts are 0.89, 0.83 and 0.73, respectively. As the theoretical molar ratio of C/N in g-C_3_N_4_ is 3/4, the correction factor between peak area ratio of C/N and molar ratio of C/N should be 1.187. Based on the correction factor, the C/N molar ratio of 0.07 V-g-C_3_N_4_ and Ce_0.07_-0.07 V-g-C_3_N_4_ can be calculated as 3/4.3 and 3/4.9, respectively, which implies the formation of N-rich carbon nitride by the incorporation of metal species. The reason for the C/N molar ratio variation may be caused by the increase of –NH groups on the g-C_3_N_4_ support surface as the metal species destroy the ordered structure of g-C_3_N_4_ partially. As reported previously, in preparation of C─N materials, the addition of metal species restrained the decomposition of nitrogen species and accelerated the decomposition of carbon species, leading to high N content in the samples [34,35]. As shown in figure 3*a*, the C 1s spectrum of g-C_3_N_4_ can be deconvoluted into three peaks with binding energies of 284.6, 285.7 and 287.5 eV, corresponding to the graphitic carbon (C─C), C─O and sp^2^ hybridized carbon (N─C═N), respectively [36]. After the incorporation of metal species, the peak intensities of both C─O and N─C═N increase, while that of C─C decreases. These imply that the rigid structural regularity of g-C_3_N_4_ was partially broken and more defects were produced on the surface of g-C_3_N_4_, generating more sites for metal species anchoring. In figure 3*b*, the N 1s spectrum of g-C_3_N_4_ shows three main peaks at 398.2, 399.3 and 400.6 eV, assigned to triazine nitrogen (C═N─C), tertiary nitrogen (N─(C)_3_) and amino function group (N─H), respectively [37]. It is worth noting that both the C 1s and N 1s peak positions of 0.07 V-g-C_3_N_4_ and Ce_0.07_-0.07 V-g-C_3_N_4_ are slightly shifted to high binding energy regions compared with those of g-C_3_N_4_. Since the graphite analogue CN matrix is believed to stabilize the metal species with the ‘coordination nest’ consisting of C and N atoms [38], it is reasonable to deduce that a strong interaction between metal species and g-C_3_N_4_ support may exist, which is reflected in the XPS study. In addition, as shown in the V 2p_3/2_ XPS spectra (figure 4), 0.07 V-g-C_3_N_4_ merely contains V^5+^ and V^4+^, while V^3+^ species appears in Ce_0.07_-0.07 V-g-C_3_N_4_. Also, the content of low oxidation state V (V^4+^, V^3+^) species increases after the addition of cerium (figure 4*b*–*d*). These may be ascribed to the reduction of partial V^5+^ species by the Ce^3+^ species during calcination [39]. In fact, similar results were reported for other Ce–V catalysts [40,41]. As listed in table 3, the (V^4+^+V^3+^)/V^5+^ ratio (calculated by the corresponding area ratio) increases gradually from 0.45 to 1.15 with the increase of cerium content, corresponding to the rise of low oxidation state vanadium species. It seems that Ce^3+^/Ce^4+^ species play an important role in maintaining the content of V^4+^ and V^3+^ species. During the oxidation of benzene to phenol over vanadium-containing catalyst using H_2_O_2_ as an oxidant, the low valent vanadium species can be oxidized to V^5+^-O-O• or V^4+^-O-O• radicals which perform as main active centres for the conversion of benzene to phenol [42]. In electronic supplementary material, figure S5, the V 2p_3/2_ XPS measurements of both 0.07 V-g-C_3_N_4_ and Ce_0.07_-0.07 V-g-C_3_N_4_ catalysts were conducted after four recycles. The recovered Ce_0.07_-0.07 V-g-C_3_N_4_ catalyst shows little change in the ratios of (V^4+^+V^3+^)/V^5+^ and V^3+^/V^4+^ relative to the fresh one, indicating that the excellent redox ability of Ce^3+^/Ce^4+^ improves the redox cycles of V^5+^/V^4+^ and V^4+^/V^3+^. Comparing with Ce_0.07_-0.07 V-g-C_3_N_4_, the recovered Ce_0.07_-0.07 V-g-C_3_N_4_ catalyst has small amount of Ce^4+^ species with the appearance of Ce^4+^ fingerprint (electronic supplementary material, figure S6) [43]. The existence of Ce^3+^/Ce^4+^ is confirmed, which promotes the formation of low state vanadium. However, •OH radicals produced by V^4+^ lead to overoxidation of benzene [44]. It is believed that V^4+^ species are more reactive than V^5+^ ones during the benzene conversion [45]. Thus a proper molar ratio of (V^4+^+V^3+^)/V^5+^ may be beneficial to achieve a good conversion of benzene and a high selectivity of phenol. Therefore, in the present case, it is reasonable to indicate that the addition of proper cerium amount improves the redox capacity of active vanadium species along with the efficient decomposition of hydrogen peroxide.

**Table 3:** The ratios of V species with different valence states on the catalyst surface.

entry	catalyst	V^4+^/V^5+^	(V^4+^+V^3+^)/V^5^	V^3+^/V^4+^
1	0.07 V-g-C_3_N_4_	0.45	0.45	—
2	Ce_0.05_-0.07 V-g-C_3_N_4_	0.53	0.93	0.78
3	Ce_0.07_-0.07 V-g-C_3_N_4_	0.62	1.09	0.75
4	Ce_0.10_-0.07 V-g-C_3_N_4_	0.64	1.15	0.74
5	0.07 V-g-C_3_N_4_ recycled	0.48	0.48	—
6	Ce_0.07_-0.07 V-g-C_3_N_4_ recycled	0.49	0.85	0.73

**Figure 5.**
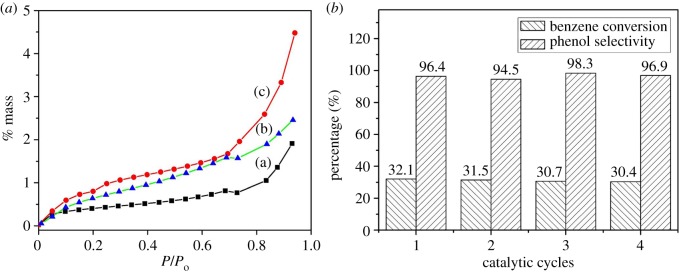
(*a*) The benzene adsorption isotherms of (a) g-C_3_N_4_, (b) 0.07 V-g-C_3_N_4_, (c) Ce_0.07_-0.07 V-g-C_3_N_4_ at 298 K. (*b*) Cyclic utilization of Ce_0.07_-0.07 V-g-C_3_N_4_.

The adsorption properties of g-C_3_N_4_, 0.07 V-g-C_3_N_4_ and Ce_0.07_-0.07 V-g-C_3_N_4_ catalysts were evaluated by studying the gravimetric uptake of benzene. In figure 5*a*, pure g-C_3_N_4_ exhibits an evident adsorption of benzene, probably attributed to the strong π–π interactions between benzene molecules and g-C_3_N_4_ [28]. Interestingly, Ce_0.07_-0.07 V-g-C_3_N_4_ exhibits the highest benzene adsorption ability among all the catalysts. As reported earlier [46,47], by increasing the content of surface basic groups (e.g. amino or hydroxyl groups) in activated carbon, the adsorption affinity for the nonpolar molecules, e.g. benzene, was enhanced. Therefore, combining with the results of XPS, it is reasonable to deduce that the increase of nitrogen-containing groups in g-C_3_N_4_ support after doping metal species can promote the benzene adsorption ability of the corresponding catalyst effectively, leading to the excellent benzene conversion.

The reusability of Ce_0.07_-0.07 V-g-C_3_N_4_ catalyst was investigated as it showed the best catalytic activity in the target reaction (figure 5*b*). After each reaction, the Ce_0.07_-0.07 V-g- C_3_N_4_ catalyst was separated, washed and dried for the next fresh reaction. After four recycles, the catalyst retained high activity without marked loss in both benzene conversion and phenol selectivity. The vanadium content of both the last recycled catalyst and the reaction solution was measured by ICP-AES, and no leaching of vanadium happened (entry 9, electronic supplementary material, table S1). In table 3, as expected, the (V^4+^ + V^3+^)/V^5+^ peak area ratio of recovered Ce_0.07_-0.07 V-g-C_3_N_4_ is 0.85, much higher than that of recovered 0.07 V-g-C_3_N_4_. These further demonstrate that the existence of Ce species facilitates the formation of V^4+^ and V^3+^ species. Moreover, XRD and FT-IR (electronic supplementary material, figures S3 and S4) measurements were conducted for the recovered Ce_0.07_-0.07 V-g-C_3_N_4_ from the fourth run. The structural properties of the recovered catalyst are nearly identical to those of fresh one, indicating the strong interactions between metal species and g-C_3_N_4_ support which accounts for high stability of Ce_0.07_-0.07 V-g-C_3_N_4_ in the direct benzene hydroxylation. The excellent stability is because of abundant defects of g-C_3_N_4_ after cerium modification that improve the dispersion and stability of V species. Although the role of V^3+^ species remains to be further studied, the work gives an insight into the design of efficient catalysts for the titled reaction in future.

A possible reaction mechanism of benzene hydroxylation is proposed based on literature work [22]. Benzene is chemically absorbed onto the surface of g-C_3_N_4_, and then the surface dispersed V^4+^ species are oxidized by H_2_O_2_ to produce V^5+^-O-O• radicals and H_2_O. The main active V^5+^-O-O• radicals are rapidly activated and react with absorbed benzene to gain the target phenol and V^5+^ is reduced to V^4+^. At the same stage, the redox V^5+^/V^4+^ is supported by Ce^3+^/Ce^4+^ to accelerate the reaction into the next recycle.

## 4. Conclusion

In this work, we developed a facile method to synthesize cerium-doped V-g-C_3_N_4_ catalysts for the direct oxidation of benzene to phenol. The dispersion of vanadium species was distinctly improved after the addition of cerium species. Among the catalysts studied, Ce_0.07_-0.07 V-g-C_3_N_4_ showed excellent catalytic performance with good reusability in the titled reaction, ascribed to the enhanced vanadium redox property, improved benzene adsorption ability and strong interactions between metal species and g-C_3_N_4_ support.

## Supplementary Material

Electronic supplementary material

## Data accessibility

The datasets supporting this article have been uploaded as part of the electronic supplementary material.
