# Supplementary material for: Cerium promoted V-g-C3N4 as highly efficient heterogeneous catalysts for the direct benzene hydroxylation
Source: R Soc Open Sci. 2018 Jun 27;5(6):180371. doi: 10.1098/rsos.180371 (PMC6030258; doi:10.1098/rsos.180371)
Supplement: Electronic supplementary material [file rsos180371supp1.doc]

**Electronic supplementary material**

**Cerium promoted V-g-C_3_N_4_ as highly efficient heterogeneous catalysts for the direct benzene hydroxylation**

Cheng Wang, Liya Hu, Meiyin Wang, Bin Yue*, Heyong He*[[1]](#footnote-2) 

*Department of Chemistry and Shanghai Key Laboratory of Molecular Catalysis and Innovative Materials, Collaborative Innovation Center of Chemistry for Energy Materials, Fudan University, Shanghai 200433, China*

**Table S1** Catalytic activity of various catalysts for benzene hydroxylation reaction.

| Entry | Catalyst | Vanadium  content  (wt.%)^a^ | Cerium content  (wt.%)^a^ | Benzene conversion  (%) | Phenol selectivity  (%) | Phenol yield  (%) | TOF^b^  value  (h^-1^) |
| --- | --- | --- | --- | --- | --- | --- | --- |
| 1 | g-C_3_N_4_ | - | - | 0.3 | 60.1 | 0.2 | - |
| 2 | C_10_H_14_O_5_V | - | - | 18.4 | 87.8 | 16.2 | - |
| 3 | 0.05Ce-g-C_3_N_4_ | - | 4.7 | 3.0 | 60.2 | 1.8 | 3.8 |
| 4 | 0.07Ce-g-C_3_N_4_ |  | 6.8 | 4.1 | 63.5 | 2.6 | 3.8 |
| 5 | 0.10Ce-g-C_3_N_4_ | - | 9.7 | 3.8 | 62.2 | 2.4 | 2.4 |
| 6 | 0.05V-g-C_3_N_4_ | 4.9 | - | 17.6 | 98.6 | 17.4 | 12.2 |
| 7 | 0.07V-g-C_3_N_4_ | 7.0 | - | 24.7 | 98.1 | 24.2 | 12.4 |
| 8 | 0.10V-g-C_3_N_4_ | 9.6 | - | 22.1 | 98.4 | 21.7 | 8.1 |
| 9 | Ce_0.07_-0.07V-g-C_3_N_4_  (the fourth round) | 6.8 | 0.3 | 30.4 | 96.9 | 29.5 | 15.6 |

Reaction condition: 1 mL of benzene, 10 mL of 80 wt% acetic acid, 40 mg of catalyst, 3.5 mL of 30 wt% H_2_O_2_, 70 ºC for 4 h.

^a^ Analyzed by ICP-AES.

^b^ Turnover frequency (TOF) was calculated as the molecules of generated phenol per metal atom per hour.


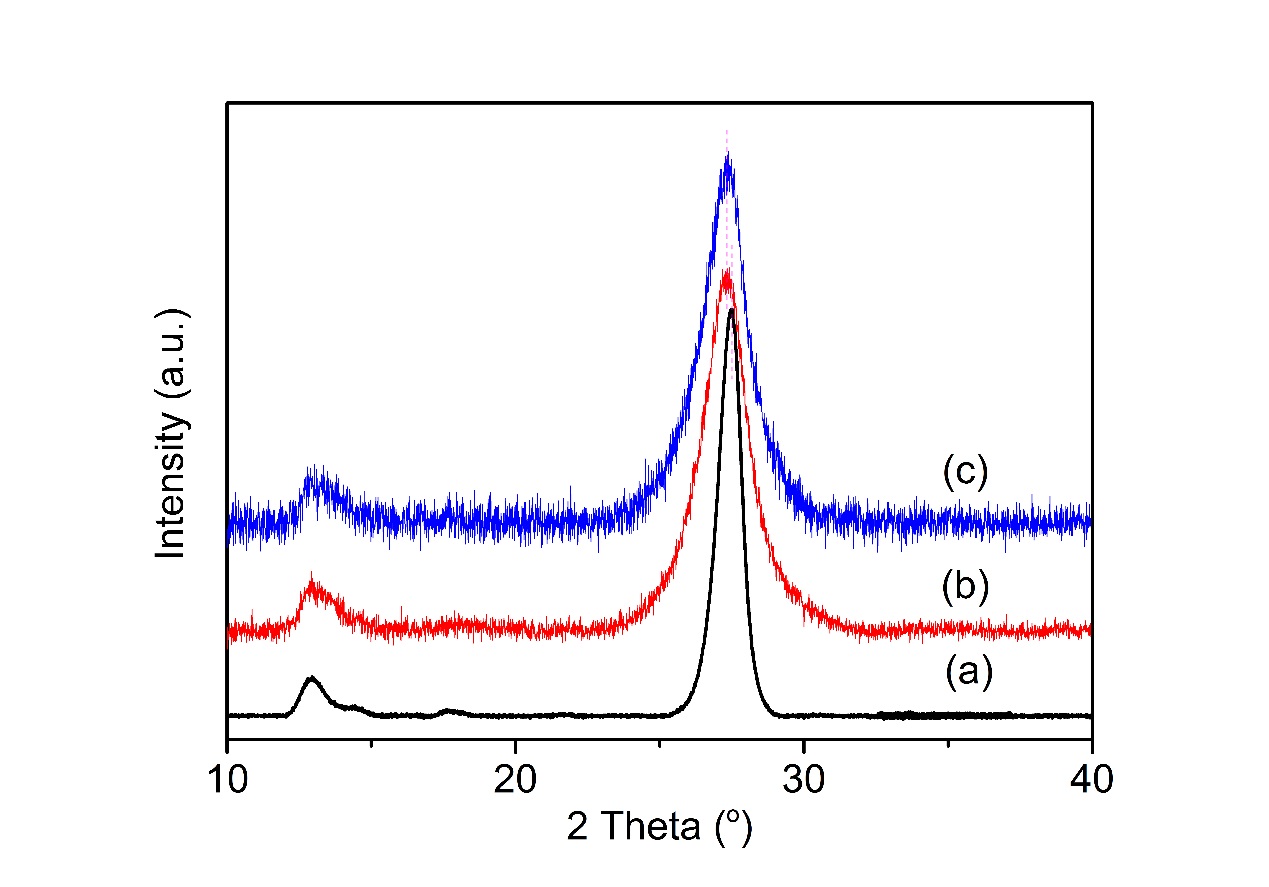


**Figure S1.** The XRD patterns of (a) g-C_3_N_4_, (b) 0.07V-g-C_3_N_4_ and (c) Ce_0.07_-0.07V-g-C_3_N_4_.


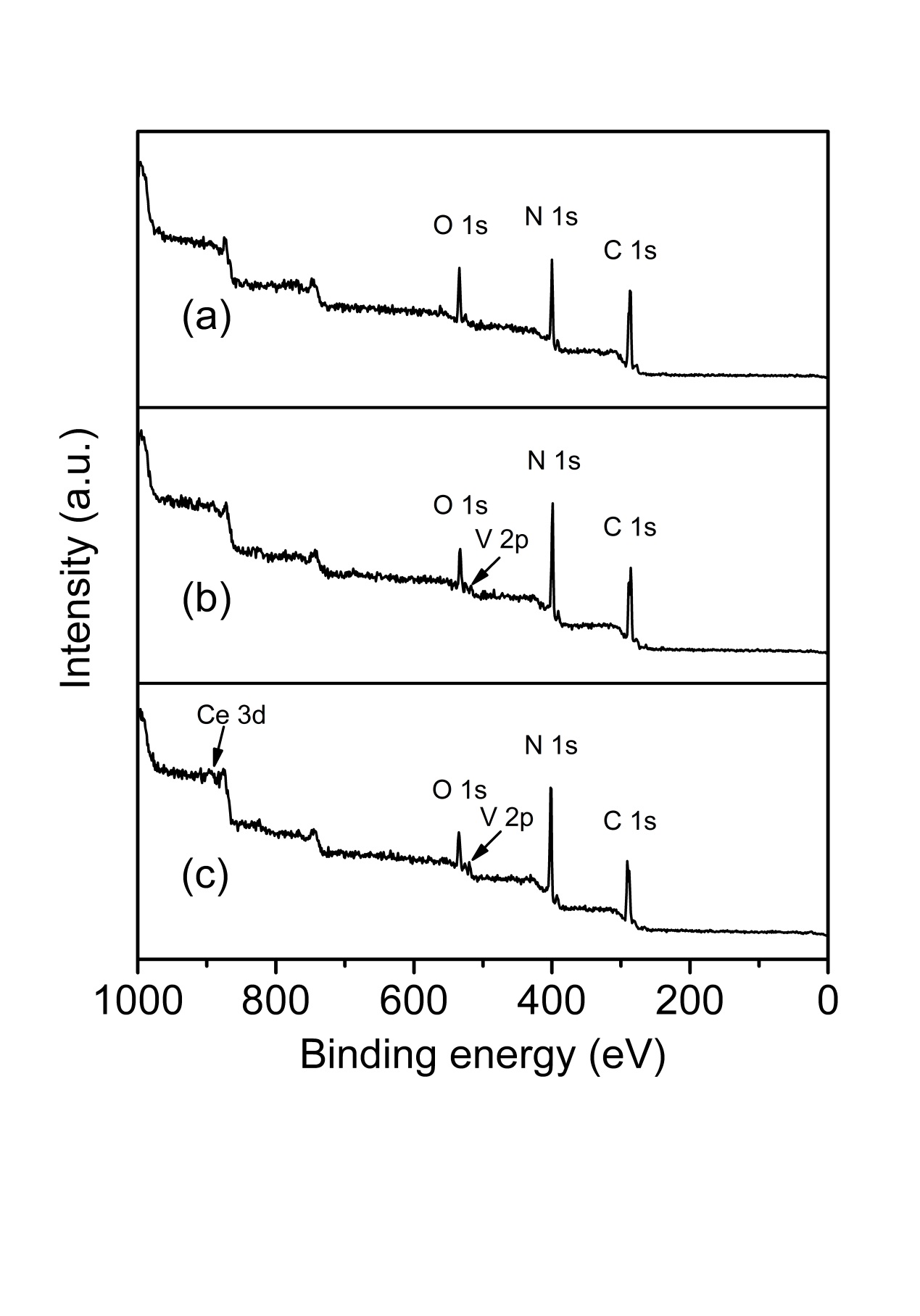


**Figure S2.** XPS survey scans of (a) g-C_3_N_4_, (b) 0.07V-g-C_3_N_4_ and (c) Ce_0.07_-0.07V-g-C_3_N_4_.


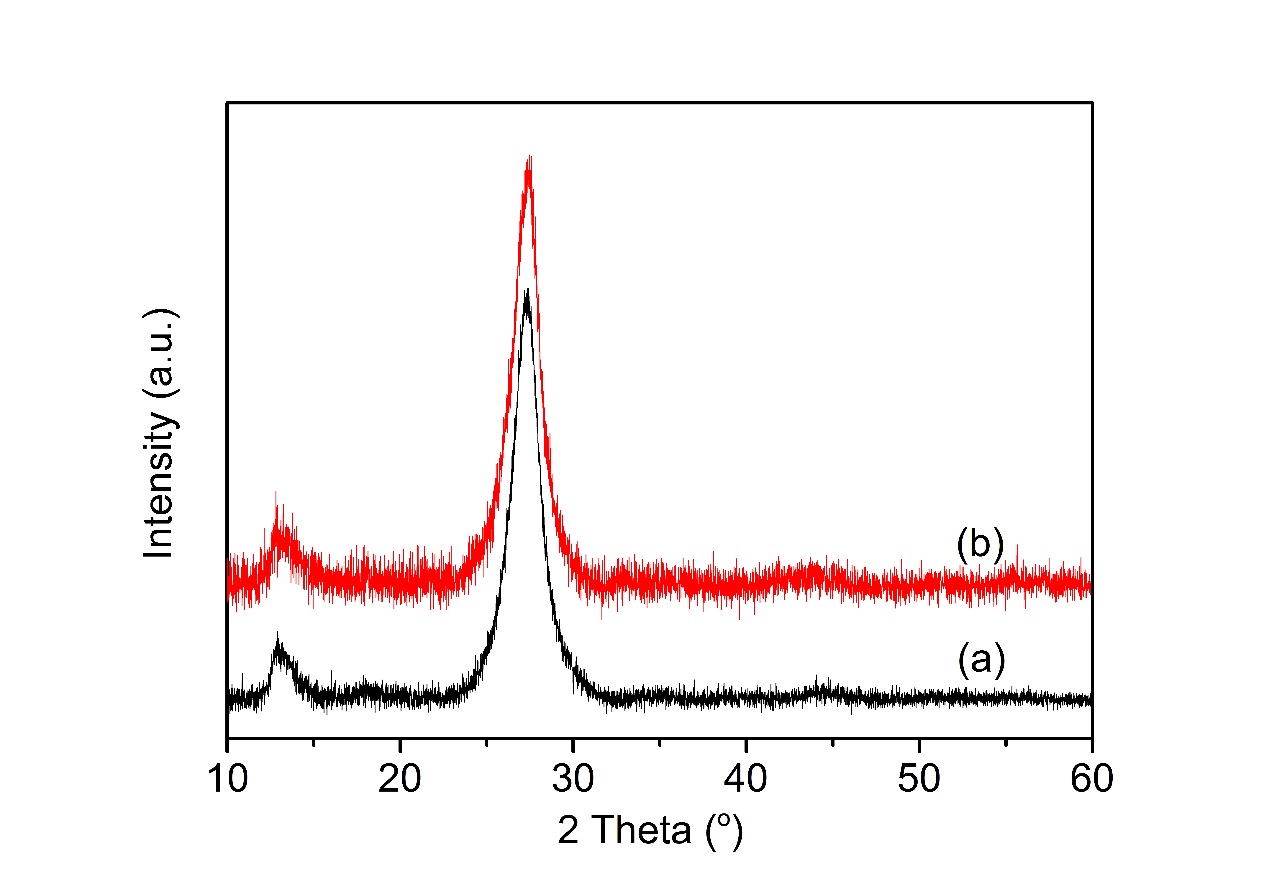


**Figure S3.** The XRD patterns of (a) Ce_0.07_-0.07V-g-C_3_N_4_ and (b) recovered Ce_0.07_-0.07V-g-C_3_N_4_ after four recycles.


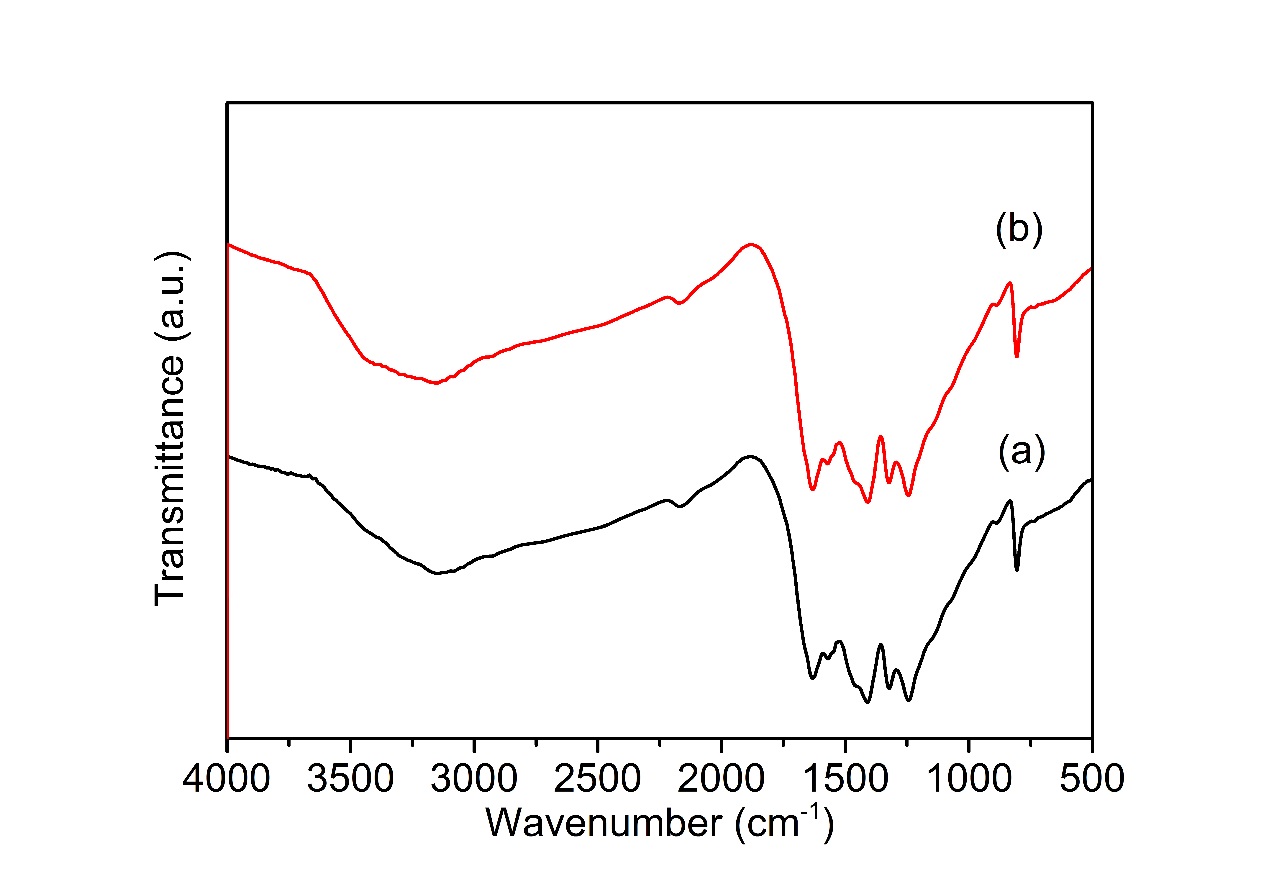


**Figure S4.** FT-IR spectra of (a) Ce_0.07_-0.07V-g-C_3_N_4_ and (b) recovered Ce_0.07_-0.07V-g-C_3_N_4_ after four recycles.


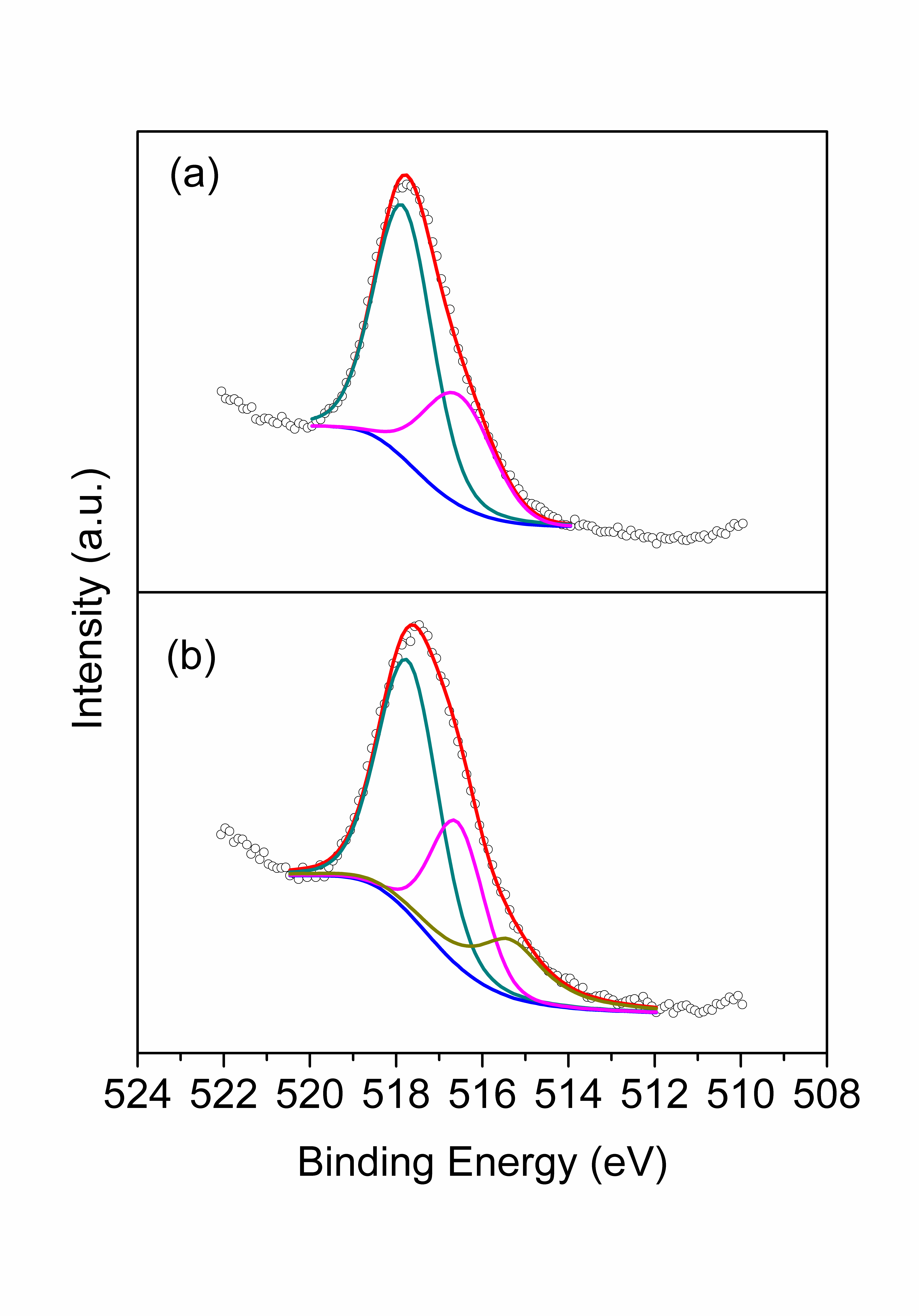


**Figure S5.** V2p_3/2_ XPS spectra of (a) recovered 0.07V-g-C_3_N_4_, (b) recovered Ce_0.07_-0.07V-g-C_3_N_4_ after four recycles.

**Figure S6.** Ce 3d_3/2_ and 3d_5/2_ XPS spectra of (a) Ce_0.07_-0.07V-g-C_3_N_4_, (b) recovered Ce_0.07_-0.07V-g-C_3_N_4_ after four recycles. The Ce 3d_3/2_ and 3d_5/2_ spectra are composed of two doublets (v and u) corresponding to the spin-orbit split 3d_5_*/*_2_ and 3d_3_*/*_2_ core holes. Four peaks corresponding to the pairs of spin-orbit doublets can be identified in the Ce 3d spectrum from Ce^3+^ species [43].

1. * Corresponding authors. Tel.: +86 21 65643916.

   E-mail addresses: yuebin@fudan.edu.cn (B. Yue), heyonghe@fudan.edu.cn (H. He). [↑](#footnote-ref-2)
